# Data sharing and the future of science

**DOI:** 10.1038/s41467-018-05227-z

**Published:** 2018-07-19

## Abstract

Who benefits from sharing data? The scientists of future do, as data sharing today enables new science tomorrow. Far from being mere rehashes of old datasets, evidence shows that studies based on analyses of previously published data can achieve just as much impact as original projects.

Data sharing has a long history in many areas of research. Although the push to encourage social and biological scientists to share and pool their results is a recent one^1^, in other fields the use of shared data has been the norm for some time. For over a century, much of economics and meteorology have been based on publicly shared data, for example.

There is a strong argument to be made that leaving data unshared is an impediment to the scientists of the future.

However, trepidation in relation to data-sharing is still prevalent in the scientific community, particularly in certain disciplines. The issues that make some researchers reluctant to share their own data have been much discussed^2^, but researchers considering using shared data as a basis for their own research also have concerns: if I want to publish high-impact work, don’t I need to collect new data? Is it the act of collecting original data that makes a study novel?

The benefits of data sharing may seem difficult to quantify. But the work of Michael P. Milham and colleagues^3^ provides direct evidence that, in the field of neuroimaging, published papers based on shared data are just as likely to appear in high-impact journals, and are just as well-cited, compared with papers presenting original data. Although citations of a manuscript and the prestige of the journal in which it appears are not direct measures of the quality or novelty of scientific output, Milham et al.’s results are likely to be reassuring for cognitive neuroscientists concerned about whether the lack of original data collection would reduce the impact of their work.

Indeed, far from being an impediment to carrying out novel science, data sharing makes new types of research possible. Consider, for instance, research using the Human Connectome Project (HCP) dataset, one of the data sharing initiatives included in the Milham et al. study. The HCP currently contains extensive fMRI, structural MRI and behavioural data from 1200 healthy young adult volunteers (https://www.humanconnectome.org/study/hcp-young-adult), and is expanding to encompass child, adolescent and older adult brains. These data are made available to any interested researcher.

While data sharing had a somewhat rocky start in the world of cognitive neuroscience^4^, the success of the HCP and the many influential studies based on it shows that its time has come. Without data sharing, it would be all but impossible for a single research group to scan 1200 people. MRI scans are expensive, and neuroimaging studies using original data typically consist of 20–50 participants. These sample sizes were sufficient to support the kinds of studies that were cutting-edge a decade ago, but today, more advanced methods require much more data.

It’s not just in neuroscience that data sharing has already transformed the kinds of studies that researchers are able to carry out. In genetics, genomics and structural biology, large shared datasets are common (e.g., ref.^5^) and many researchers have used and re-used previously published datasets to enable new discovery in these areas^6^.

In the physical sciences, data sharing is also increasingly practiced. In astronomy and astrophysics, for example, telescope data is typically open;^7^ without such sharing, most research groups, lacking the funds to construct the kinds of large telescopes required for modern astronomy research, would be unable to reach the cutting edge of discovery. Astronomy data sharing has even expanded to encompass personal computers with the UC Berkeley-based SETI@home program, enabling citizen science participation in data analysis^8^.

The field of ecology has made tremendous strides thanks to data sharing under the USA’s Long-Term Ecological Research (LTER) Network^9^. This network, a set of long-running observations across different ecosystems, has allowed ecologists to detect important patterns playing out over timescales exceeding the length of research appointments or funding cycles. The extent of data sharing in the field more broadly has evolved over time^10^ but influential publications are now arising more than ever from databases supported by large networks of researchers^11^.

These examples demonstrate one clear benefit of data sharing, in that it enables individual researchers to punch above their financial weight by making large, or expensive-to-collect, datasets available to all. In this way, data sharing opens hence unforeseen avenues of research. This is not just true of large-scale data sharing initiatives: even relatively small datasets, if shared, can contribute to big data and fuel future scientific discoveries in unexpected ways. In medicine, for example, the patient-level meta-analysis of large number of past clinical trials has revealed numerous novel findings that go well beyond the original purpose of the studies that generated the data (e.g., ref.^12^).

Sharing data, then, is not only a way to improve the reproducibility and robustness of the science that is taking place today^13^, but can drive new science for tomorrow. Given that we today cannot predict how valuable a given set of data will one day prove to be, there is a strong argument to be made that leaving data unshared is an impediment to the scientists of the future. Indeed, we can envision a time in which, far from being a disruptive innovation, data sharing is seen as a normal and essential part of the scientific process, much the way we see peer-review.

While SETI@home hasn’t found any aliens intelligence just yet, there are billions of stars in our galaxy: how else would we reach for the stars unless we aim together where alone? While neuroscientists haven’t yet solved the mysteries of human brain even using shared data, with some 86 billion neurons^14^ in a single brain, they will need to work together to cover them all.
